# Two‐year impact of COVID‐19: Longitudinal MRI brain changes and neuropsychiatric trajectories

**DOI:** 10.1111/pcn.13789

**Published:** 2025-02-04

**Authors:** Ravi Dadsena, Julia Walders, Ana S. Costa, Sophie Wetz, Sandro Romanzetti, Stella Andrea Lischewski, Christina Krockauer, Josephine Heine, Lars Schlenker, Pia Klabunn, Katia Schwichtenberg, Tim J. Hartung, Christiana Franke, Carolin Balloff, Ferdinand Binkofski, Jörg B. Schulz, Carsten Finke, Kathrin Reetz

**Affiliations:** ^1^ Department of Neurology RWTH Aachen University Aachen Germany; ^2^ JARA Brain Institute Molecular Neuroscience and Neuroimaging (INM‐11) Research Centre Jülich and RWTH Aachen University Aachen Germany; ^3^ Charité‐Universitätsmedizin Berlin Department of Neurology and Experimental Neurology Berlin Germany; ^4^ Humboldt‐Universität zu Berlin Faculty of Philosophy, Berlin School of Mind and Brain Berlin Germany; ^5^ Department of Neurology, Medical Faculty and University Hospital Düsseldorf Heinrich Heine University Düsseldorf Germany; ^6^ Department of Neurology Kliniken Maria Hilf GmbH Mönchengladbach Germany; ^7^ Division for Clinical Cognitive Sciences, Department of Neurology RWTH Aachen University Aachen Germany; ^8^ Research Center Jülich GmbH Institute for Neuroscience and Medicine (INM‐4) Jülich Germany

**Keywords:** fatigue, longitudinal study, neuroimaging, post‐COVID‐19 condition

## Abstract

**Aim:**

Up to 10% of SARS‐CoV‐2 infected individuals suffer from post‐COVID‐19 condition, marked by fatigue and cognitive dysfunction as major symptoms. Longitudinal studies on neuropsychological and clinical trajectories and related brain changes are scarce. Here, we aimed to examine their evolution up to 2 years post‐infection.

**Methods:**

In a multi‐center, longitudinal study of 79 post‐COVID patients (mean age 46, 48 female) with persistent symptoms and 21 age‐ and sex‐matched never‐infected, healthy controls (mean age 42, eight female), we analyzed neuropsychological performance, self‐reported outcomes and associated neuroimaging alterations of resting‐state functional and structural magnetic resonance imaging data 23 months post‐infection.

**Results:**

In post‐COVID patients 23 months after SARS‐CoV‐2 infection we observed (1) that fatigue severity had reduced but still remained present in most patients, (2) widespread brain changes involving the brainstem, the pre‐ and postcentral gyrus and the limbic olfactory network, (3) a weakening of self‐reported fatigue and its cerebral associations. Notably, findings of brain aberrations were more pronounced in hospitalized patients.

**Conclusion:**

Our findings indicate that complex brain adaptations take place up to 2 years following SARS‐CoV‐2 infection. Some regions manifest enduring abnormalities while others undergo restitution. The attenuation of radio‐clinical associations suggests a compensatory function for these regions, pointing to non‐brain intrinsic factors to sustain persistent fatigue.

Four years following the onset of the COVID‐19 pandemic, our understanding of the persistent effects of a SARS‐CoV‐2 infection remains limited. Even 18 months after COVID‐19 infection, 10% of individuals continue to experience one or more symptoms that could be linked to the virus.^1^ Long COVID describes persistent or newly developing symptoms 4 weeks and post‐COVID‐19 condition 3 months after COVID‐19. Fatigue, cognitive dysfunction and dyspnea constitute the major symptom burden. Long‐term studies reported a high prevalence of fatigue and cognitive deficits up to 2 years after initial SARS‐CoV‐2 infection.^2^, ^3^, ^4^ Despite intensive research efforts, a comprehensive pathophysiological framework sufficiently explaining the multisystemic effects and, specifically, the high occurrence of neurological symptoms of post‐COVID‐19 condition is still missing. Current hypotheses include immune system impairment, microbiome alterations, autoimmune processes, coagulopathy, a dysfunctional vagus nerve and/or brainstem, and psychosomatic factors.^5^, ^6^, ^7^ In an earlier study, we found that visible cerebral lesions are absent in the majority of cases except for cerebral microbleeds, which were primarily related to extracorporeal membrane oxygenation during acute COVID‐19.^8^ Meanwhile, numerous brain imaging studies investigated microstructural, metabolic, and functional changes in the central nervous system.^9^, ^10^, ^11^, ^12^, ^13^ Although varying in severity and clinical importance most of these cross‐sectional neuroimaging studies provide strong evidence, that brain abnormalities are linked to self‐reported postviral symptoms. Frequently affected brain regions in post‐COVID patients include limbic structures, the olfactory cortex, the hippocampus, the basal ganglia, the orbitofrontal cortex, the thalamus, the brainstem, and the cerebellum.^9^, ^10^, ^11^, ^12^, ^14^, ^15^ Longitudinal neuroimaging studies are scarce. The longitudinal landmark study from the UK Biobank compared MRI scans before and after SARS‐CoV‐2 infection. 4.7 months post‐SARS‐CoV‐2 infection regions with secondary connections to the olfactory cortex, such as the orbitofrontal cortex and the parahippocampal gyrus, exhibit increased gray matter thickness and reduced tissue contrast in MRI scans.^15^ This pattern persists in a comprehensive multimodal imaging study conducted 11 months after COVID‐19. In this study, diminished connectivity between the left and right parahippocampal regions and the orbitofrontal and cerebellar areas are accompanied by a reduction in gray matter volume in cortical, limbic, and cerebellar regions.^11^

Longitudinal clinical and neuroimaging studies are warranted to determine the temporal evolution of these brain changes, to identify whether they are permanent or dynamic and to elucidate how they manifest on a clinical level. In the present multi‐center study, we conduct a multimodal imaging analysis to evaluate longitudinal structural and functional brain changes in post‐COVID patients up to 2 years after SARS‐CoV‐2 infection, and to determine their clinical relation to major neurological post‐COVID symptoms including fatigue and cognitive problems. Building on the results of our previous neuroimaging study conducted 7 months after COVID‐19 in post‐COVID patients, we hypothesize that changes in brain regions associated with the limbic olfactory network and brainstem will remain evident 2 years after infection.^14^ Considering the typical clinical improvement observed in post‐COVID patients, we aim to investigate whether these brain regions show signs of recovery.

## Materials and Methods

### Study participants

The study was conducted in two university hospitals in Germany (Aachen and Berlin). At both sites, patients were prospectively recruited from the Department of Neurology. Imaging data from healthy controls without any record of neurological or psychiatric conditions were sourced from prior research projects before the onset of the COVID‐19 pandemic utilizing identical imaging protocols and with a similar time period between scans as included patients (Table S1). Participants eligible for the study were 18 years or older with persistent neurological symptoms after confirmed SARS‐CoV‐2 infection, as determined by reverse transcription polymerase chain reaction of nasopharyngeal swab or the presence of antibodies against SARS‐CoV‐2, with no prior vaccination. Magnetic resonance imaging (MRI) exclusion criteria included contraindications like metallic implants or claustrophobia. Hospitalization during acute COVID‐19 was used to determine disease severity. The study included 79 participants and 21 age‐ and sex‐matched healthy controls for analysis. Approval for all procedures was obtained from the local ethics committees (“Ethikkommission an der Medizinischen Fakultät der RWTH Aachen”, EK 192/20, and “Ethikkommission der Charité – Universitätsmedizin Berlin”, EA2/007/21) and followed the Declaration of Helsinki. All individuals gave written informed consent before participation in the study.

### Procedures

#### Clinical measures

As previously outlined standardized patient reported outcome measures (PROMS) administered at both sites and at both timepoints included: the Fatigue Scale for Motor and Cognitive Functions (FSMC), the Hospital Anxiety and Depression Scale (HADS‐D), the Epworth Sleepiness Scale (ESS), and the Pittsburgh Sleep Quality Index (PSQI).^8^, ^9^

The FSMC is a 20‐item self‐report scale, using a 5‐point Likert scale and distinguishing between motor and cognitive fatigue symptoms with a maximum score of 100.^16^ The overall score and subscales include cut‐offs for mild (≥43), moderate (≥53), and severe fatigue (≥63).

For HADS‐D, the maximum score includes 21 for anxiety and depression, respectively. Severity thresholds were considered for each subscale: ≤7 for normal, 8–10 for questionable, and ≥10 for increased.^17^ ESS and PSQI measured symptoms of sleep disorders.

For ESS, category 0–7 indicates unlikely abnormal sleepiness, 8–9 suggests an average amount of daytime sleepiness, 10–15 implies potential excessive sleepiness, and 16 up to the maximum of 24 signals excessive sleepiness requiring medical attention. For PSQI the maximum is 21 whereas a cut‐off value of five distinguishes “good” and “poor” quality sleepers. The individual symptom evolution over time was assessed using a questionnaire at the Berlin site and through a structured interview at the Aachen site.

#### Neuropsychological assessment

A neuropsychological assessment was conducted at both timepoints. Montreal Cognitive Assessment (MoCA) was used as a brief screening tool. The neuropsychological assessment protocol included common standardized measures of attention, information processing and psychomotor speed (Trail Making Test‐A, TMT‐A; Alertness subtests of the Test of Attentional Performance, TAP), executive functions (Stroop Test, phonemic and semantic verbal fluency, Digit span backward, TMT‐B), language (Naming, phonemic and semantic verbal fluency), visuospatial processing (Rey‐Osterrieth Complex Figure copy, ROCFT), memory and learning (Complex Figure delayed recall, Digit span forward, Rey Auditory Verbal Learning Test, RAVLT). Protocols were identical at both sites except for a different version of the Stroop Test, and a parallel version of the phonemic and semantic verbal fluency test for follow‐up analysis used at the Aachen site. Impairment was characterized as a performance falling below the 16th percentile rank (PR) or between −2 and −1.5 for the z‐score, and severe impairment was designated as a PR below 2 or a z‐score below −2.^18^ For the c‐score, mild impairment was defined below 3 and severe impairment below 1. These classifications were determined based on published normative data, adjusted for age, education, and/or sex, depending on the specific test.

#### MR imaging data acquisition

MRI data collection was carried out uniformly at both research sites at baseline and follow‐up utilizing PRISMA whole‐body scanners with a 3T field strength from Siemens (Erlangen, Germany) (Table S1). High‐resolution *T*_1_‐weighted anatomical scans and functional imaging sequences were performed during the resting state condition. During the functional scan, ambient light was reduced and participants were advised to keep their eyes open without focusing on specific thoughts, aiming to capture intrinsic brain activity without directed tasks or stimuli.

#### Multimodal imaging analysis

**Fig. 1:**
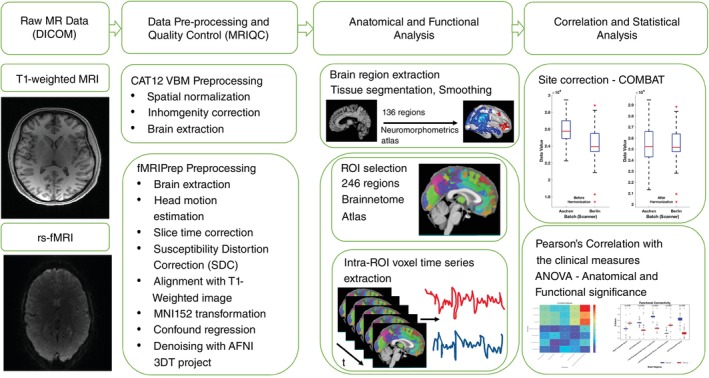
Overview of pipeline workflow. Shown are steps and respective components regarding multimodal neuroimaging analyses including data pre‐processing and quality controls, anatomical and functional analysis and correlation and statistical analyses.

The methodological approach for multimodal imaging analysis is represented in Fig. 1. Initially, the imaging data were structured into the Brain Imaging Data Structure format. Subsequently, raw *T*_1_‐weighted anatomical scans and resting‐state functional scans were subjected to a thorough quality check using MRIQC (v.23.1.0).^19^ Following the quality control assessments, imaging data were visually examined for potential artifacts. For functional data, subjects were excluded if motion framewise displacement (mFD) exceeded 0.30 mm. Furthermore, a two‐tiered strategy identified gross motion outliers (>0.55 mm of mFD), with stricter criteria for exclusion: (i) mFD >0.30 mm; (ii) more than 20% of the FDs exceed 0.2 mm; or (iii) any FDs >5 mm.^20^ Participants who met the established quality control criteria proceeded to further processing. All 79 subjects were included for baseline and follow‐up structural analysis whereas functional analysis included 65 participants at baseline and 62 at follow‐up.

The rsfMRI data was preprocessed using fMRIPrep (v.23.1.3), a Nipype (v.1.8.6) based program in Python (v.3.7.1).^21^ The preprocessing pipeline involved motion and slice‐time correction, co‐registration, computation of confounding time‐series (FD, DVARS, global signals). Physiological regressors for component‐based noise correction were derived using temporal and anatomical variants. Nuisance time‐series were derived through principal components analysis, and finally, the BOLD time‐series were resampled to (Montreal Neurological Institute (MNI)152NLin2009cAsym) space. Detailed functional preprocessing steps are outlined in the supplement (Text S1).

The 3dTproject tool from the Analysis of Functional NeuroImages (v.23.2.08) was used to denoise the preprocessed rsfMRI data.^22^ This includes eliminating 12 motion parameters and anatomical component‐based noise correction components created from the first five white matter and five cerebrospinal fluid parameters given by fMRIPrep, as previously mentioned. This approach effectively removes the effects of head motion, cardiac, and respiratory noise.^23^ After denoising, a Gaussian kernel with a full width at half maximum of 6 mm was used to smooth the fMRI data.

##### Functional MRI data

Brain functional connectivity patterns were extracted from preprocessed data using the Brainnetome atlas and exploratory Region of Interest (ROI) analysis. This comprehensive atlas, featuring 246 subregions derived from fMRI data across both hemispheres, yielded detailed insights into brain subregion connectivity. The initial step involved aligning the Brainnetome atlas with the MNI152 space, ensuring spatial alignment across participants for comparative analysis. Subsequently, subject‐specific masks were generated based on the atlas to represent various brain sub‐regions of interest. Using the FMRIB Software Library (v.6.0.6.5) package *via* a bash script, contrast estimates were derived from individual functional data using these masks as templates. Following this, z‐scores were extracted from each of the 246 masks representing labeled brain areas for each subject post‐contrast estimation.^24^ Finally, a comprehensive statistical and correlation analysis was performed on these z‐scores.

##### Structural MRI data

We used the Computational Anatomy Toolbox (CAT12) longitudinal model with neurodevelopment effects (http://www.neuro.uni-jena.de/cat/index.html) within the software packages Statistical Parametric Mapping 12 (Wellcome Department of Cognitive Neurology, UCL, London, United Kingdom) software package to preprocess longitudinal structural MRI data and undertake Voxel Based Morphometry in MATLAB (v.R2022a). Initially, intrasubject images were realigned. The reference image was created from the mean subject image for successive realignment and bias correction to correct for signal inhomogeneities and motion‐related artifacts. Then, the images were segmented into gray matter, white matter, and cerebrospinal fluid and normalized to the MNI space. Successively, the total intracranial volume for each subject was estimated and used as a covariate in later modeling. To process the images further, smoothing was done. The gray matter images were smoothed specifically with the 8 mm full‐width at half‐maximum smoothing kernel, as recommended from the CAT12 package. The group comparisons were performed using preprocessed and normalized gray matter maps with mean values extracted from various regions using the Neuromorphometrics atlas for further statistical analysis.^25^

### Hospitalization and fatigue differences

Analyses were carried out separately excluding 15 patients who were hospitalized with acute COVID‐19. Of these 15 patients, eight required admission to the intensive care unit, and within this group, five had been on mechanical ventilation. Furthermore, we observed severe fatigue in 36 participants during follow‐up, as indicated by their FSMC scores. To identify any specific brain abnormalities in this subgroup, we exclusively analyzed those with severe fatigue at follow‐up.

### Statistical analysis

Functional and structural imaging datasets were preprocessed using the ComBat approach, an empirical Bayesian method for data harmonization, to reduce potential confounding effects caused by site‐specific changes, that is scanner effects across data collecting sites.^26^ The normality of the functional, anatomical, and clinical datasets was evaluated using the Shapiro–Wilk test at a significance level of alpha = 0.05 to ensure adherence to parametric and non‐parametric test assumptions. Subsequently, datasets that passed the Shapiro–Wilk test underwent analysis using one‐way Analysis of Variance (ANOVA) followed by Tukey's *post hoc* test. Conversely, datasets that did not pass the normality test were subjected to the Kruskal‐Wallis test, followed by Dunn's *post hoc* test for multiple comparisons. We also employed two‐way ANOVA to investigate the interaction effects of two main factors: group (consisting of healthy controls and COVID‐19 patients) and timepoints (including baseline and follow‐up data).^27^ Variance homogeneity was assessed using the F‐test. Significance thresholds were set at *P* < 0.05, *P* < 0.01, and *P* < 0.001, indicating two‐sided significance tests. Mean and standard deviation were computed and reported accordingly. To supplement our analysis, we calculated effect sizes (Omega‐squared for ANOVA), which provided information about the magnitude of the differences observed: small (0.01), medium (0.06), and large (0.14).^28^ Additionally, we used a combination of data removal and imputation techniques to address missing data (Text S2).^29^ Furthermore, we applied Pearson's correlation coefficient analyses, setting the significance level at *P* < 0.05, to examine the relationships between structural and functional connectivity metrics and clinical parameters, as well as neuropsychological scores. Statistical analyses were conducted using Python (v.3.7.1).

## Results

### Clinical and demographic characteristics of study participants

Characteristics of study participants are shown in Table 1. The cohort of 79 participants included predominantly women (61%) and had a mean age of 46.4 ± 11.3 years at the baseline visit. Except for one individual with epilepsy, patients had no history of relevant neurological disorders. Mean months since acute COVID‐19 were 7.0 ± 4.2 for baseline and 23.2 ± 4.0 for the follow‐up visit. There were no significant differences in age, sex or education when comparing patients and healthy controls. Fatigue, dyspnea and headache were the most frequently reported symptoms at follow‐up visit (Fig. S1). Five study participants reported to have fully recovered with no ongoing symptoms.

**Table 1:** Clinical and demographic characteristics of all study participants

	Baseline		Follow‐up	
	Post‐COVID‐19	Healthy controls	Post‐COVID‐19	Healthy controls
Site Aachen/Berlin T1	49/30	21/0	49/30	21/0
Site female/male rs‐fMRI	37/28	19/0	36/26	16/0
Sex female/male	48/31	8/13	48/31	8/13
Hospitalization (ICU/with ventilation)	15 (8/5)		15 (8/5)	
Age	46.43 ± 11.28	40.63 ± 14.54	47.02 ± 11.06	41.63 ± 15.45
Days since diagnosis	210.6 ± 128.03		697.53 ± 120.23	
Cardiovascular risk factors^a^	29			
Neurological comorbidities^b^	15			
Psychiatric comorbidities^c^	7			
MoCA	27 ± 2		26 ± 3	
FSMC total	64 ± 19		61 ± 24	
FSMC cognitive	33 ± 10		30 ± 12	
FSMC motor	31 ± 10		30 ± 12	
HADS total	13 ± 7		11 ± 8	
HADS anxiety	7 ± 4		6 ± 4	
HADS depression	6 ± 4		5 ± 4	
ESS	10 ± 6		9 ± 5	
PSQI	10 ± 4		9 ± 5	

*Note*: Values are mean ± standard deviation except for site, sex, hospitalization and comorbidities where counts are reported. All study participants were age‐ and sex‐matched.

Abbreviations: ESS, Epworth Sleepiness Scale; FSMC, Fatigue Scale for Motor and Cognitive functions; HADS, Hospital Anxiety and Depression Scale; ICU, intensive care unit; MoCA, Montreal cognitive Assessment; PSQI, Pittsburgh Sleep Quality Index; rs‐fMRI, resting state functional magnetic resonance imaging.

^a^ Including arterial hypertension, diabetes mellitus type II, dyslipidemia, overweight.

^b^ Meningioma (*n* = 1), mild head trauma (*n* = 1), migraine (*n* = 9), epilepsy (*n* = 1).

^c^ Depression (*n* = 4), condition after burn out (*n* = 2), anxiety disorder (*n* = 1), post‐traumatic stress disorder (*n* = 1).

### Neuropsychological assessment and symptom questionnaires

**Fig. 2:**
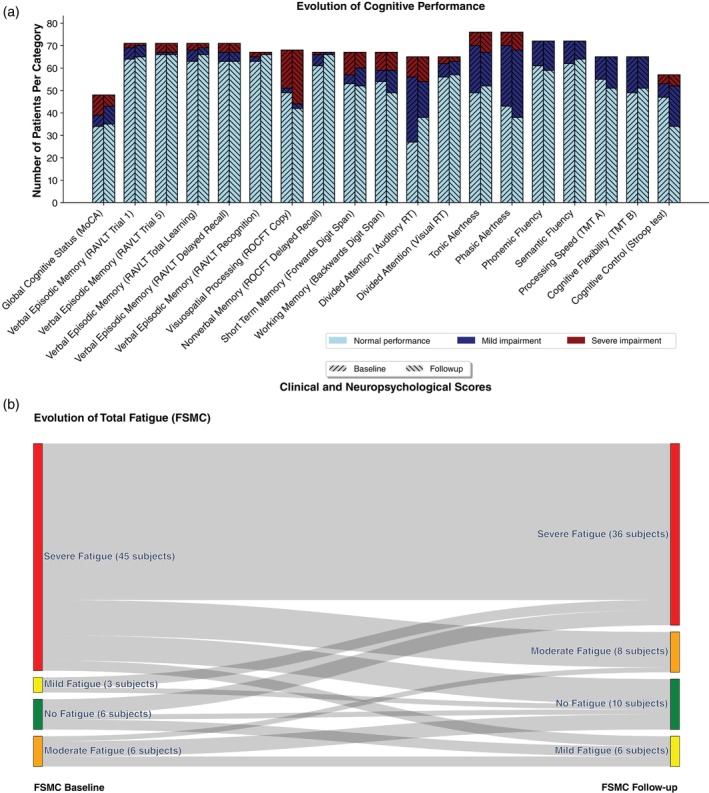
Cognitive impairment and evolution of self‐reported fatigue. (a) Barplots indicate the number of patients with severe impairment, mild impairment and normal cognitive performance adjusted for age, sex and education, where applicable at baseline and follow‐up analyses. Only data where test results were available at both time points are shown. MoCA, Montreal Cognitive Assessment; RAVLT, Rey Auditory Verbal Learning Test; ROCFT, Rey‐Osterrieth complex figure copy; RT, Response time; TMT, Trail Making Test. (b) Sankey‐diagram illustrating the evolution of self‐assessed overall fatigue with the Fatigue Scale for Motor and Cognitive Functions (FSMC). Only data where the FSMC was available at both time points (*n* = 60) are shown.

In most cognitive domains performance was within the normal range (Fig. 2a). Exceptions included divided attention and alertness, with cognitive impairment affecting between one third and nearly half of the patients. There was an overall trend for improvement especially regarding divided attention, and cognitive flexibility, although measures of phasic alertness, visuospatial processing, cognitive control and processing speed worsened in some individuals (Table S2).

A range of neuropsychological measures and PROMS showed significant correlations at both visits. Amongst others, we found the overall FSMC to be significantly correlated with the HADS‐D depression score (baseline: *r* = 0.42, follow‐up: *r* = 0.41), the TMT A (baseline: *r* = 0.36, follow‐up: *r* = 0.37) and the TMT B (baseline: *r* = 0.31, follow‐up: *r* = 0.34). Self‐assessed neuropsychiatric questionnaires during baseline visit indicated the presence of severe fatigue (FSMC mean 64 ± 19) equally affecting cognitive and physical functions, increased daytime sleepiness (ESS mean 10 ± 6), and poor sleep quality (PSQI mean 10 ± 4), while there were no indications for abnormal depressive (HADS mean 6 ± 4) or anxiety (HADS mean 7 ± 4) symptoms. Results on these measures remained stable at follow‐up with no significant change. However, there was an overall trend for improvement of self‐reported fatigue in a subset of study participants (Fig. 2b).

### Structural and functional brain changes

**Fig. 3:**
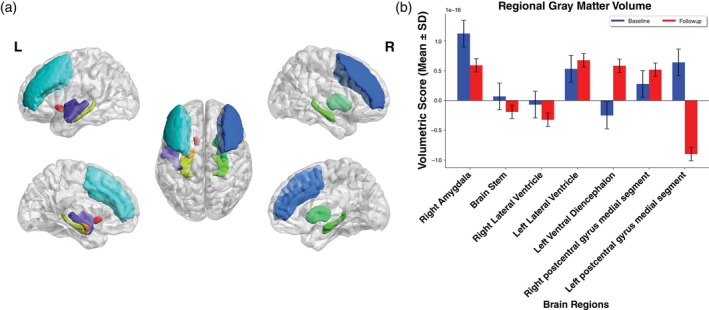
Longitudinal structural brain changes in post‐COVID patients. (a) Shown are all eight brain regions with significant volumetric differences between baseline and follow‐up analysis in post‐COVID patients across lateral, medial and dorsal view. (b) The bar plot indicates the change of the volumetric scores of respective brain regions.

Structural brain changes at baseline and follow‐up visits are shown in Fig. 3a,b. Compared to healthy controls, volumetric analyses at baseline showed a significantly lower volume of the right postcentral gyrus in post‐COVID patients. Longitudinal analyses in post‐COVID patients revealed a total of eight brain regions that significantly changed over time. Amongst others, a volumetric increase was found in the left lateral ventricle and the right postcentral gyrus whereas the brainstem, the right lateral ventricle, and the left postcentral gyrus decreased in volume. The same significant changes in these regions also became evident when comparing healthy controls to post‐COVID patients at follow‐up. Information on effect sizes and *P*‐values are shown in Table S3. In longitudinal structural brain imaging analyses of healthy controls, a volume reduction was also detectable in the left postcentral gyrus (*P* < 0.001, ω^2^ = 0.03) and in the brainstem (*P <* 0.05, *ω* ^2^ = 0.22). Our analyses on interaction effects yielded no significant results.

**Fig. 4:**
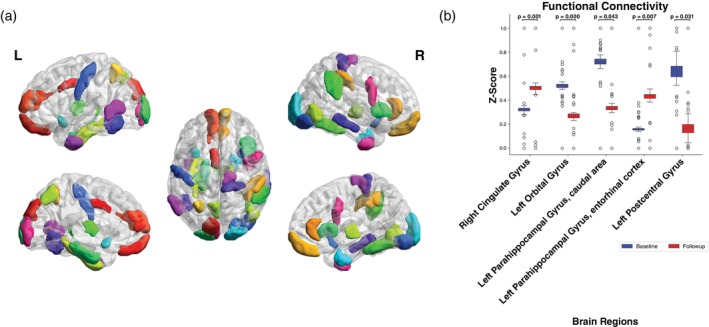
Longitudinal functional brain changes in post‐COVID patients. (a) Shown are all 35 brain regions with significant functional connectivity differences between baseline and follow‐up analysis in post‐COVID patients from ROI‐ROI analysis across lateral, medial, and dorsal views. (b) The box plot shows five out of the 35 brain regions with significant correlations in more than three neuropsychological measures at baseline and follow‐up analysis.

Main results of functional analyses are depicted in Fig. 4a,b. In our baseline functional network analyses, we identified 20 brain regions with an altered connectivity in post‐COVID patients compared to healthy controls including regions in the parietal, occipital, frontal, and temporal lobe. Amongst others, the frontal orbital gyrus, the frontal precentral gyrus, and the parahippocampal gyrus displayed changes in functional connectivity. In post‐COVID patients, longitudinal fMRI analyses revealed 35 brain regions that showed alterations across various areas of the brain amongst which 17 regions showed a decreased connectivity and 18 an increase in connectivity. When comparing healthy controls with post‐COVID patients only six of these brain changes also showed significant changes at follow‐up. Information on effect sizes and *P*‐values are shown in Table S4. In contrast, healthy controls only had 10 affected brain regions located in the frontal, parietal, and occipital lobes. Information on interaction effects is reported in the supplement (Text S3).

### Correlations of neuropsychological assessments and brain changes

**Fig. 5:**
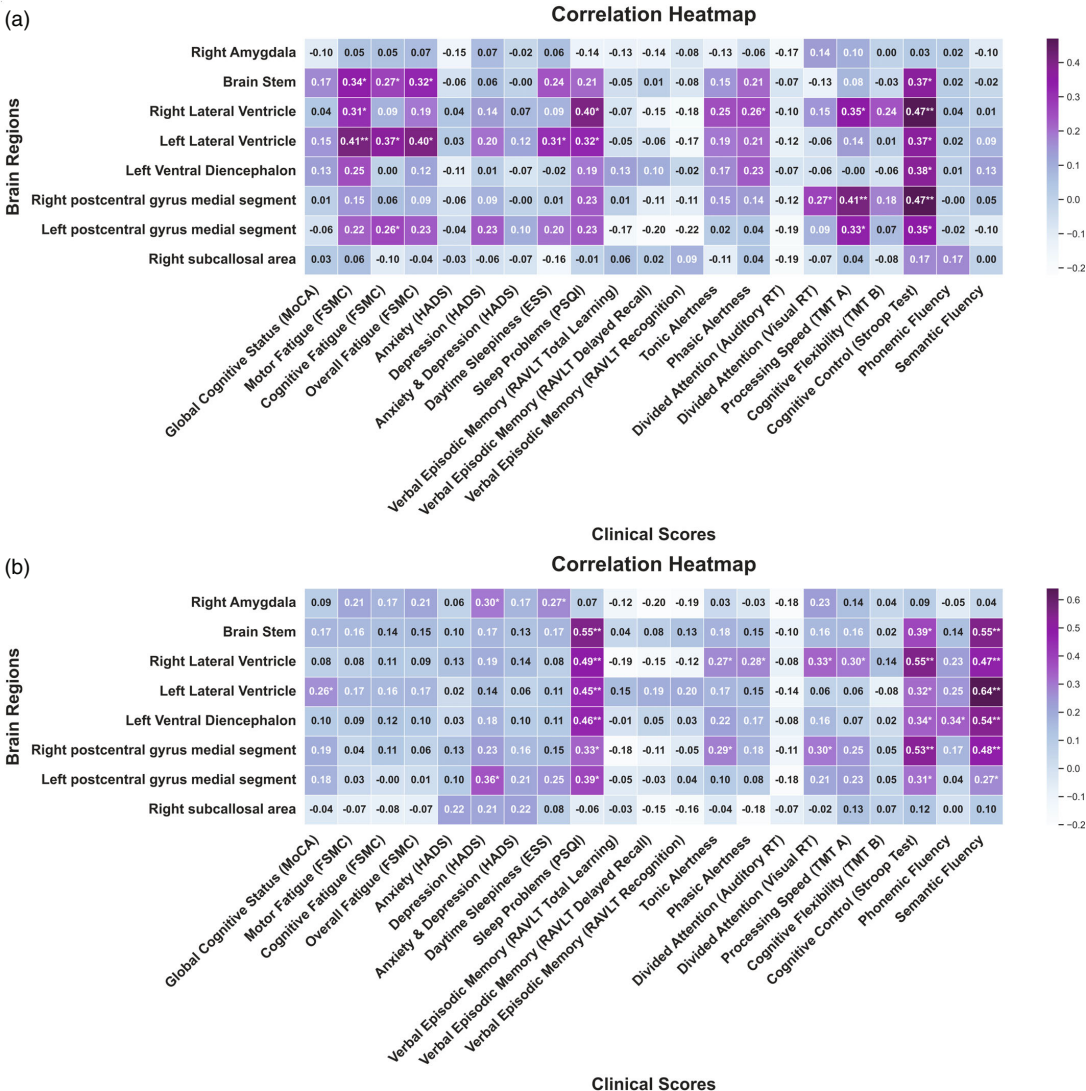
Correlation heatmaps of structurally altered brain regions with neuropsychological and clinical measures in post‐COVID patients. Visualization of a heatmap plot depicting Pearson's correlation coefficients between all structurally altered brain regions and corresponding neuropsychological and clinical measures for post‐COVID patients for (a) baseline correlation coefficient and (b) follow‐up correlation coefficient. Positive coefficients signify a positive association, while negative coefficients denote an inverse relationship between the variables. MoCA, Montreal Cognitive Assessment; FSMC, Fatigue Scale for Motor and Cognitive Functions; HADS, Hospital Anxiety and Depression Scale; ESS, Epworth Sleepiness Scale; PSQI, Pittsburgh Sleep Quality Index; RT, Response time; TMT, Trail Making Test.

Here, we report only significant correlations. For baseline structural imaging parameters, we found positive correlations between the overall FSMC and the brainstem (*r* = 0.32) (Fig. 5a). Processing speed was positively correlated with the left (*r* = 0.33) and right (*r* = 0.41) postcentral gyrus. In follow‐up analyses, positive correlations were found for the volume of the right amygdala and the HADS‐D (*r* = 0.30). The PSQI showed positive correlations across various brain regions including the brainstem (*r* = 0.55) (Fig. 5b). Semantic fluency and cognitive control showed positive correlations with most altered brain regions, except for two.

**Fig. 6:**
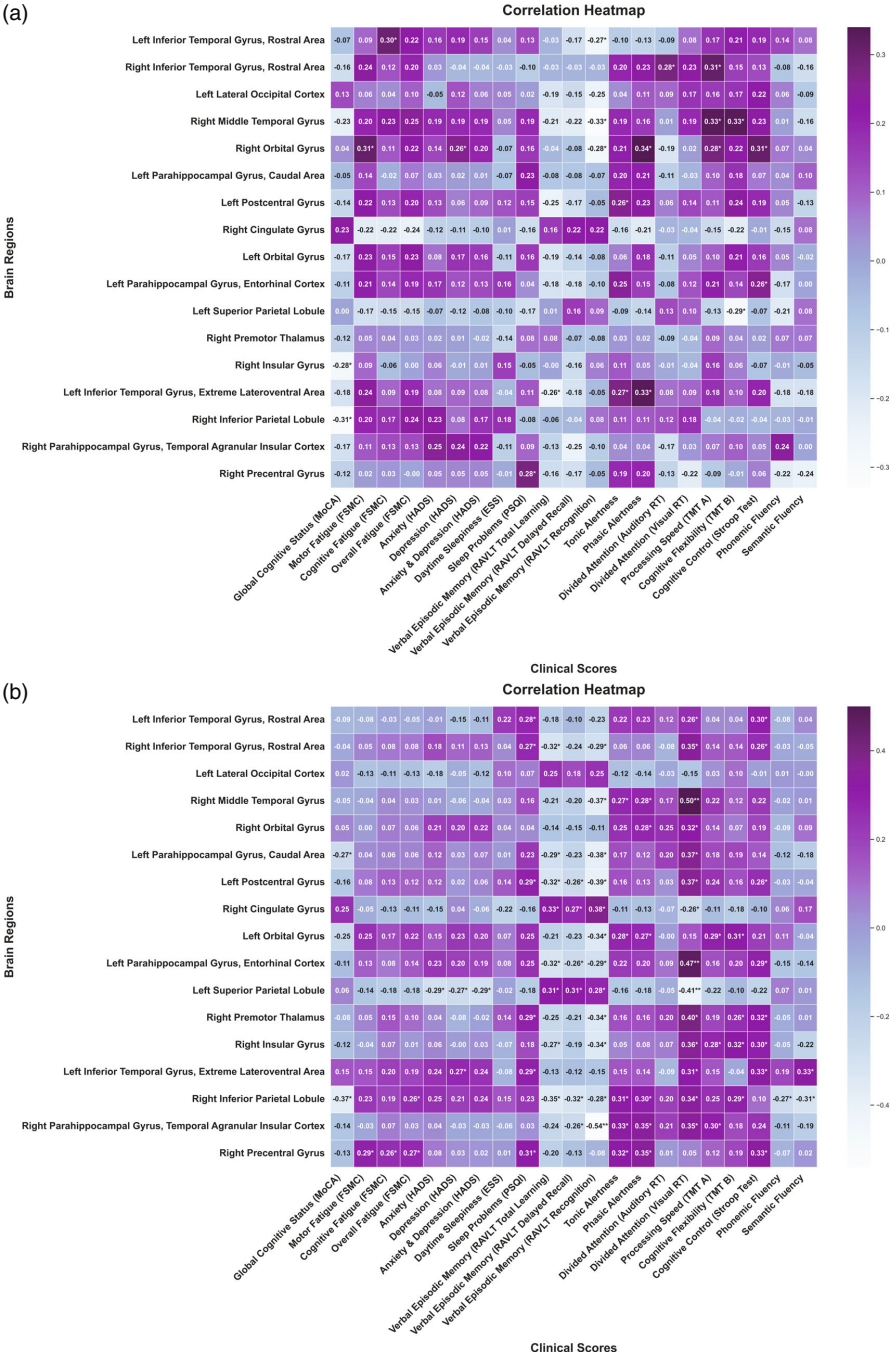
Correlation heatmaps of functionally altered brain regions with neuropsychological and clinical measures in post‐COVID patients. Visualization of a heatmap plot depicting Pearson's correlation coefficients between specific resting state fMRI brain regions and corresponding neuropsychological and clinical measures for post‐COVID patients for (a) baseline correlation coefficient and (b) follow‐up correlation coefficient. Positive coefficients signify a positive association, while negative coefficients denote an inverse relationship between the variables. ESS, Epworth Sleepiness Scale; FSMC, Fatigue Scale for Motor and Cognitive Functions; HADS, Hospital Anxiety and Depression Scale; MoCA, Montreal Cognitive Assessment; PSQI, Pittsburgh Sleep Quality Index; RT, Response time; TMT, Trail Making Test.

For functional imaging parameters a link between fatigue ratings and network aberrations in brain areas including the basal ganglia, temporal lobe, frontal lobe, and insular lobe was found at baseline (Fig. 6a). However, except for the right precentral gyrus (*r* = 0.27) and right inferior parietal lobule (*r* = 0.26), no evident network association with fatigue was observed during follow‐up (Fig. 6b). Divided visual attention showed significant correlations across a wide range of functionally altered brain regions.

### Hospitalization and fatigue differences

Excluding hospitalized individuals, most findings on longitudinal brain alterations in key regions remained the same. However, the increase in size of the left lateral ventricle, as a marker for general cerebral atrophy, was not significant in the non‐hospitalized group. Also, certain areas including the insula, cuneus gyrus, parahippocampal gyrus, and postcentral gyrus showed no significant connectivity changes in this group. One region in the left orbital regions (*P <* 0.05, *ω* ^2^ = 0.98) and one in the inferior parietal lobule (*P <* 0.05, *ω* ^2^ = 0.99) were only significantly altered in the non‐hospitalized sample.

Thirty‐six study participants reported severe fatigue at follow‐up assessment. Compared to the remaining study participants, there was no decrease in the volume of the right amygdala noticeable in these patients. Functionally, 19 regions showed significantly changed connectivity, and eight regions distinguished them from the remaining cohort. Amongst others, these included regions in the orbital gyrus, the precentral gyrus, and the thalamus.

## Discussion

Our multimodal longitudinal clinical and brain imaging study of 79 post‐COVID patients showed that nearly half reported persistent severe fatigue, and nearly one third experienced cognitive impairments in attention, alertness, executive function, and visuospatial processing up to 23 months after infection, although there was an overall trend for improvement. These symptoms were accompanied by a widespread longitudinal network and structural reorganization compared to age‐ and sex‐matched healthy controls. Brain areas with longitudinal changes involved the brainstem, the pre‐ and postcentral gyrus and the limbic olfactory network. Remarkably, while structural and functional brain abnormalities linked to fatigue decreased over time, the cognitive effects remained. In line with our results, recent studies have shown measurable cognitive deficits lasting up to a year and ongoing fatigue for up to 20 months after COVID‐19.^2^, ^12^, ^30^, ^31^

Brain connectivity changes are physiological to a certain extent,^32^ however, compared to healthy controls in a similar time frame, the number of regions undergoing structural and network changes was much higher in post‐COVID patients. These alterations were balanced in terms of directionality, with both increases and decreases in volume and cerebral connectivity. This suggests reorganizational structural and functional adaptations rather than a progressive downward trend. Structurally, the brainstem was linked to fatigue at baseline and showed a significant volume decrease in post‐COVID patients. This aligns with the strong network disruptions related to fatigue found in our earlier study 7 months after mild SARS‐CoV‐2 infection.^14^ Given its key role in regulating consciousness and alertness, the autonomic system and several other vital body functions, the brainstem could play a major role in driving fatigue. Adding to this, a multicenter study found a visually detectable pattern of brainstem hypometabolism in post‐COVID patients^10^ and a recent study using 7T MRI found long‐term microstructural abnormalities in the medulla oblongata, pons, and midbrain around 6.5 months post‐hospitalization.^33^ The brainstem has recently been identified as a region exhibiting significant vulnerability to the inflammatory damage associated with acute COVID‐19 infection.^34^ This vulnerability may contribute to the pathogenesis of long‐term neurological sequelae. Peripheral serotonin deficiency found after viral infection could lead to reduced vagus nerve signaling in post‐COVID patients and thereby further add to its dysfunction.^35^

Strikingly, despite persistent fatigue reported by study participants, its initial associations with brainstem alterations and network changes weakened substantially over time. This points to non‐neural factors that contribute to or maintain fatigue in the long‐term course. While the etiology of chronic post‐COVID fatigue is unknown, findings from Legler *et al*. suggest that immune‐triggered vascular dysregulation may contribute to persistent severe fatigue in patients who also meet the criteria for chronic fatigue syndrome.^2^

Psychosomatic and psychiatric factors may also contribute to persistent symptoms as clinical studies have consistently shown that pre‐existing neuropsychiatric conditions significantly increase the risk and impact of post‐acute COVID‐19 symptoms.^4^, ^6^, ^7^, ^36^

Lastly, fatigue assessment tools, such as the FSCM used here, may lack the sensitivity to detect changes in fatigue frequency. This is supported by a study of 1022 post‐COVID patients showing decreased self‐reported fatigue frequency but only reduced severity, and not frequency, according to the FSCM,^37^ highlighting the need for biomarkers of fatigue.

Regarding cognitive function, we found widespread associations between most structurally changed brain areas and executive functioning assessed by the Stroop test and semantic fluency at follow‐up. Interestingly, we observed an increase in volume on the right and a decrease on the left post central gyrus, both positively correlating with measures of executive function. This suggests interhemispheric compensation, wherein reduced right gyrus volume at baseline may be functionally offset by left‐hemisphere activity. Subsequent volume recovery in the right gyrus at follow‐up suggests a process of neural restitution and may indicate restoration of intrinsic function. In line with this, Capelli *et al*. also report COVID‐related gray matter reduction in the postcentral gyri at different degrees of severity.^38^ Cortical atrophy in the postcentral gyrus as well as lower functional connectivity in the precentral gyrus was previously found to be associated with fatigue in chronic fatigue syndrome and multiple sclerosis.^39^, ^40^ Due to its anatomical location, it would be a well accessible target for transcranial magnetic stimulation, which was shown to improve fatigue in post‐COVID patients.^41^ Similarly to the increase and decrease of volume of the postcentral gyri, the changes in the lateral ventricles as measures for more general cerebral atrophy could indicate a recovery process.

Regarding connectivity changes, the regions were widespread across the brain, with some focus on the memory‐relevant limbic olfactory network in line with previous studies.^13^, ^15^, ^42^ These included the parahippocampal gyrus, the orbital gyrus and the cingulate gyrus. Several of these regions showed correlations with fatigue, particularly the orbital gyrus and the left inferior temporal gyrus, which tended to weaken over time except in the precentral gyrus. In this region, brain changes emerged as more pronounced compared to less fatigued individuals.

The partially differing patterns of brain changes between hospitalized and non‐hospitalized patients suggest distinct stages of brain vulnerability and recovery, further supporting a link between COVID‐19 severity and persistent neurological effects beyond the resolution of acute symptoms. Additionally, our study further supports the notion that fatigue and cognitive impairment are two distinct sequelae of COVID‐19 with potentially distinct pathophysiological pathways as their cerebral associations develop distinctly.^7^

The major strengths of the study are the long follow‐up duration, the multicentric character, and the state‐of‐the‐art multimodal imaging analyses combined with a comprehensive and standardized neuropsychological testing battery. The present study has some limitations. First, neuroimaging or cognitive data from study participants before their SARS‐CoV‐2 infection were not available within this study limiting our knowledge on pre‐existing aberrations. Our structural analyses revealed significant longitudinal changes in the ventricles, postcentral gyri, and brainstem of post‐COVID patients. These changes were also apparent when comparing the post‐COVID group to healthy controls at follow‐up, further supporting their contribution to the long‐term sequelae of COVID‐19. However, some of the brain changes identified here may be unrelated to post‐COVID‐19 pathogenesis. A study conducted by Hosp *et al*. compared asymptomatic post‐COVID‐19 individuals with healthy controls, revealing persistent alterations in the brainstem, cerebellum and corpus callosum even in the absence of overt symptoms.^43^ This suggests that not all observed changes are functionally relevant, or that distinct pathogenic mechanisms, for example inflammation *versus* degeneration, may underlie the observed alterations.

Additionally, neuropsychological testing and assessments of PROMS was not performed in healthy controls and they were not specifically recruited for this study. Although study protocols at Berlin and Aachen were harmonized, we cannot exclude possible bias regarding differences in protocols and procedures.

This study demonstrates an overall trend towards improvement in both cognitive function and self‐reported fatigue 2 years post‐SARS‐CoV‐2 infection, although a significant proportion of patients continued to experience fatigue. Concurrent with this clinical improvement, evidence of complex compensatory network reorganization was observed. These findings suggest that while acute SARS‐CoV‐2 infection may induce lasting alterations in brain structure, network integrity, and metabolism, the persistence of symptoms may be significantly modulated by extra‐neural factors such as inflammatory processes, autoimmune responses, and psychosocial factors. Long‐term longitudinal studies focused on tracking organic and functional changes are essential to fully elucidate the pathophysiological mechanisms underlying persistent post‐COVID‐19 symptoms.

## Disclosure statement

No conflict of interest involved in the study.

## Author contributions

Conceptualization: KR, CFi, JW, ASC. Investigation: JW, ASC, SL, SR, SW, CK (Aachen site). JH, KS, TH, CFi, PK, LS, CFr (Berlin site). Resources: JBS, FB (Aachen site). CFi (Berlin site). Formal analysis: RD, CB. Writing‐original draft: JW, RD. Visualization: RD, JW. Writing – review & editing: all authors.

## Supporting information


**Data S1.** Supporting Information.
**Text S1.** Detailed information of functional preprocessing pipeline.
**Text S2.** Detailed information on handling and removing data.
**Text S3.** Information on interaction effects.
**Table S1.** Scanning parameters for high‐resolution T1‐weighted anatomical scan and functional imaging sequence.
**Table S2.** Percentage of post‐COVID patients with impairment of cognitive performance for each test and visit.
**Table S3.** Comparative analysis of statistically significant structural brain changes in matched healthy controls (HC) and post‐COVID patients: Baseline and follow‐up assessments with adjusted *P*‐values and omega‐squared effect sizes (*ω* ^2^).
**Table S4.** Comparative analysis of statistically significant functional brain regions: Baseline and follow‐up in matched healthy Controls (HC) and post‐COVID patients with adjusted *P*‐values and omega‐squared effect Sizes (*ω* ^2^).

## Data Availability

Deidentified imaging, clinical and neuropsychological data are available on request from the corresponding author.
