# Supplementary material for: Two‐year impact of COVID‐19: Longitudinal MRI brain changes and neuropsychiatric trajectories
Source: Psychiatry Clin Neurosci. 2025 Feb 4;79(4):176–86. doi: 10.1111/pcn.13789 (PMC11962352; doi:10.1111/pcn.13789)
Supplement: Supplementary file 1 — Data S1. Supporting Information. Text S1. Detailed information of functional preprocessing pipeline. Text S2. Detailed information on handling and removing data. Text S3. Information on interaction effects. Table S1. Scanning parameters for high‐resolution T1‐weighted anatomical scan and functional imaging sequence. Table S2. Percentage of post‐COVID patients with impairment of cognitive performance for each test and visit. Table S3. Comparative analysis of statistically significant structural brain changes in matched healthy controls (HC) and post‐COVID patients: Baseline and follow‐up assessments with adjusted P‐values and omega‐squared effect sizes (ω 2). Table S4. Comparative analysis of statistically significant functional brain regions: Baseline and follow‐up in matched healthy Controls (HC) and post‐COVID patients with adjusted P‐values and omega‐squared effect Sizes (ω 2). [file PCN-79-176-s001.docx]

**Supplementary Material**

[1. Methods 1](#_Toc164694491)

[**Text S1. Detailed information of functional preprocessing pipeline** 1](#_Toc164694492)

[**Text S2. Detailed information on handling and removing data** 4](#_Toc164694493)

[**Text S3. Information on interaction effects.** 4](#_Toc164694494)

[2. Tables 4](#_Toc164694495)

[**Table S1. Scanning parameters for high-resolution T1-weighted anatomical scan and functional imaging sequence.** 4](#_Toc164694496)

[**Table S2. Percentage of post-COVID patients with impairment of cognitive performance for each test and visit.** 4](#_Toc164694497)

[**Table S3. Comparative analysis of statistically significant structural brain changes in matched healthy controls (HC) and post-COVID patients: Baseline and follow-up assessments with adjusted p-values and omega-squared effect sizes (ω²).** 5](#_Toc164694498)

[**Table S4. Comparative analysis of statistically significant functional brain regions: Baseline and follow-up in matched healthy Controls (HC) and post-COVID patients with adjusted p-values and omega-squared effect Sizes (ω²).** 5](#_Toc164694499)

[3. Figures 7](#_Toc164694500)

[4. References 7](#_Toc164694501)

# **1. Methods**

## **Text S1. Detailed information of functional preprocessing pipeline**

**Preprocessing of B_0_ inhomogeneity mappings**
For Berlin data, the B0-nonuniformity map (or fieldmap) was estimated based on two (or more) echo-planar imaging (EPI) references with topup; FSL None) whereas for the Aachen dataset the fieldmap was estimated from the phase-drift map(s) measure with two consecutive GRE (gradient-recalled echo) acquisitions (Andersson et al., 2003). The corresponding phase-map(s) were phase-unwrapped with prelude (FSL None).

**Structural data preprocessing**

A total of 1 T1-weighted (T1w) images were found within the input BIDS dataset. The T1-weighted (T1w) image was corrected for intensity non-uniformity (INU) with N4BiasFieldCorrection (Tustison et al., 2010), distributed with antsRegistration ANTs (v.23.2.08) (Avants et al., 2008), and used as T1w-reference throughout the workflow.The T1w-reference was then skull-stripped with a Nipype implementation of the antsBrainExtraction.sh workflow (from ANTs), using OASIS30ANTs as target template. A total of 1 T1-weighted (T1w) images were found within the input BIDS dataset. The T1-weighted (T1w) image was corrected for intensity non-uniformity (INU) with N4BiasFieldCorrection (Tustison et al., 2010), distributed with ANTs (version unknown) (Avants et al., 2008), (RRID:SCR_004757), and used as T1w-reference throughout the workflow. The T1w-reference was then skull-stripped with a *Nipype* implementation of the antsBrainExtraction.sh workflow (from ANTs), using OASIS30ANTs as target template. Brain tissue segmentation of cerebrospinal fluid (CSF), white-matter (WM) and gray-matter (GM) was performed on the brain-extracted T1w using fast (FSL (v.6.0.6.5) (Zhang et al., 2001). Brain surfaces were reconstructed using recon-all (FreeSurfer 7.3.2, RRID:SCR_001847) (Dale et al., 1999), and the brain mask estimated previously was refined with a custom variation of the method to reconcile ANTs-derived and FreeSurfer-derived segmentations of the cortical gray-matter of Mindboggle (Klein et al., 2017). Volume-based spatial normalization to one standard space (MNI152NLin2009cAsym) was performed through nonlinear registration with ANTs, using brain-extracted versions of both T1w reference and the T1w template. The following template were selected for spatial normalization and accessed with TemplateFlow (23.0.0) (Ciric et al., 2017): ICBM 152 Nonlinear Asymmetrical template version 2009c (Fonov et al., 2009) [TemplateFlow ID: MNI152NLin2009cAsym].

**Functional data preprocessing**

For each functional BOLD run (across all sessions), the following preprocessing steps were implemented. First, a reference volume and its skull-stripped version were generated using a custom methodology of fMRIPrep. Head-motion parameters with respect to the BOLD reference (transformation matrices, and six corresponding rotation and translation parameters) are estimated before any spatiotemporal filtering using mcflirt (FSL) (Jenkinson et al., 2002). The estimated fieldmap was then aligned with rigid-registration to the target EPI (echo-planar imaging) reference run. The field coefficients were mapped on to the reference EPI using the transform. BOLD runs were slice-time corrected to 1.06s (0.5 of slice acquisition range 0s-2.13s) using 3dTshift from AFNI (Cox & Hyde, 1997) (RRID:SCR_005927). The BOLD reference was then co-registered to the T1w reference using bbregister (FreeSurfer) which implements boundary-based registration (Greve & Fischl, 2009). Co-registration was configured with six degrees of freedom. Several confounding time-series were calculated based on the preprocessed BOLD: framewise displacement (FD), DVARS and three region-wise global signals. FD was computed using two formulations following Power (absolute sum of relative motions, (Power et al., 2014) and Jenkinson (relative root mean square displacement between affines, (Jenkinson et al., 2002)). FD and DVARS are calculated for each functional run, both using their implementations in Nipype (following the definitions by Power et al. 2014) (Power et al., 2014). The three global signals are extracted within the CSF, the WM, and the whole-brain masks. Additionally, a set of physiological regressors were extracted to allow for component-based noise correction (CompCor) (Behzadi et al., 2007). Principal components are estimated after high-pass filtering the preprocessed BOLD time-series (using a discrete cosine filter with 128s cut-off) for the two CompCor variants: temporal (tCompCor) and anatomical (aCompCor). tCompCor components are then calculated from the top 2% variable voxels within the brain mask. For aCompCor, three probabilistic masks (CSF, WM and combined CSF+WM) are generated in anatomical space. The implementation differs from that of Behzadi et al. in that instead of eroding the masks by 2 pixels on BOLD space, a mask of pixels that likely contain a volume fraction of GM is subtracted from the aCompCor masks (Behzadi et al., 2007). This mask is obtained by dilating a GM mask extracted from the FreeSurfer’s aseg segmentation, and it ensures components are not extracted from voxels containing a minimal fraction of GM. Finally, these masks are resampled into BOLD space and binarized by thresholding at 0.99 (as in the original implementation). Components are also calculated separately within the WM and CSF masks. For each CompCor decomposition, the k components with the largest singular values are retained, such that the retained components’ time series are sufficient to explain 50 percent of variance across the nuisance mask (CSF, WM, combined, or temporal). The remaining components are dropped from consideration. The head-motion estimates calculated in the correction step were also placed within the corresponding confounds file. The confound time series derived from head motion estimates and global signals were expanded with the inclusion of temporal derivatives and quadratic terms for each (Satterthwaite et al., 2013). Frames that exceeded a threshold of 0.5 mm FD or 1.5 standardized DVARS were annotated as motion outliers. Additional nuisance timeseries are calculated by means of principal components analysis of the signal found within a thin band (crown) of voxels around the edge of the brain, as proposed by Patriat et al. (Patriat et al., 2017). The BOLD time-series were resampled into standard space, generating a preprocessed BOLD run in MNI152NLin2009cAsym space. First, a reference volume and its skull-stripped version were generated using a custom methodology of fMRIPrep. All resamplings can be performed with a single interpolation step by composing all the pertinent transformations (i.e. head-motion transform matrices, susceptibility distortion correction when available, and co-registrations to anatomical and output spaces). Gridded (volumetric) resamplings were performed using antsApplyTransforms (ANTs), configured with Lanczos interpolation to minimize the smoothing effects of other kernels (Lanczos, 1964). Non-gridded (surface) resamplings were performed using mri_vol2surf (FreeSurfer). The presented description was generated automatically by fMRIPrep. which operates under the CC0 license and then modified by the authors of this work for clarity and accuracy.

## **Text S2. Detailed information on handling and removing data**

To ensure data quality and reliability, all subjects were systematically excluded using predefined inclusion criteria. Following the extraction of brain regions from structural and functional data, a median-based imputation method was used to address missing values for specific regions. This method was selected as it can provide a reliable estimate of missing values without adding significant bias to the dataset. We used a selective approach to analyze the clinical and neuropsychological data. Only subjects who had test results for specific clinical and neuropsychological scores were included in the plotting and analysis stages.

## **Text S3. Information on interaction effects.**

Our analyses on interaction effects regarding functional imaging analyses yielded significant results in six brain regions including the left cingulate gyrus, caudodorsal area (*p =* 0.004, ω^2^ = 0.74 ), the left inferior parietal lobe, rostroventral area, (*p =* 0.007, ω^2^ = 0.71), the right occipital lateral superior gyrus (*p =* 0.046, ω^2^ = 0.57), the right parahippocampal gyrus, caudal area (*p =* 0.028, ω^2^ = 0.62), the right superior frontal gyrus, lateral area (*p =* 0.003, ω^2^ = 0.75), and the right lateral, prefrontal thalamus (*p =* 0.005, ω^2^ = 0.73).

# **2. Tables**

**Table S1. Scanning parameters for high-resolution T1-weighted anatomical scan and functional imaging sequence.**

|  | **Aachen** | | **Berlin** | |
| --- | --- | --- | --- | --- |
| Parameter | T1 | EPI | T1 | Multi-Band EPI |
| Scanner | Siemens Prisma | | Siemens Prisma Fit | |
| Field Strength (T) | 3 | | 3 | |
| Dimensions | 208×288×288 | 64×64×36 | 191×215×200 | 104×104×72 |
| Volumes | n/a | 205 | n/a | 720 |
| Voxel Resolution (mm) | 0.8 | 3.1×3.1×3.6 | 1 | 2 |
| Echo Time (ms) | 2.36 | 30 | 2.64 | 37 |
| Repetition Time (s) | 2.4 | 2.21 | 2.5 | 0.8 |
| Flip Angle (°) | 9 | 90 | 8 | 52 |

## **Table S2. Percentage of post-COVID patients with impairment of cognitive performance for each test and visit.** Abbreviations: MoCA = Montreal Cognitive Assessment, RAVLT = Rey Auditory Verbal Learning Test, ROCFT = Rey-Osterrieth Complex Figure copy, RT = Response time, TMT = Trail Making Test.

| Clinical and Neuropsychological Scores | **﻿Degree of cognitive impairment** | | | |
| --- | --- | --- | --- | --- |
|  | **﻿Mild impairment (%)** | | **Severe impairment (%)** | |
|  | **Baseline** | **Follow-up** | **Baseline** | **Follow-up** |
| Global Cognitive Status (MoCA) | 10.42 | 16.67 | 18.75 | 10.42 |
| Verbal Episodic Memory (RAVLT Trial 1) | 7.04 | 7.04 | 2.82 | 1.41 |
| Verbal Episodic Memory (RAVLT Trial 5) | 1.41 | 1.41 | 5.63 | 5.63 |
| Verbal Episodic Memory (RAVLT Total Learning) | 7.04 | 4.23 | 4.23 | 2.82 |
| Verbal Episodic Memory (RAVLT Delayed Recall) | 5.63 | 5.63 | 5.63 | 5.63 |
| Verbal Episodic Memory (RAVLT Recognition) | 2.99 | .. | 2.99 | 1.49 |
| Visuospatial Processing (ROCFT Copy) | 2.94 | 2.94 | 25.00 | 35.29 |
| Nonverbal Memory (ROCFT Delayed Recall) | 7.46 | 1.49 | 1.49 | .. |
| Short Term Memory (Forwards Digit Span) | 5.97 | 11.94 | 14.93 | 10.45 |
| Working Memory (Backwards Digit Span) | 7.46 | 14.93 | 11.94 | 11.94 |
| Divided Attention (Auditory RT) | 44.62 | 24.62 | 13.85 | 16.92 |
| Divided Attention (Visual RT) | 9.23 | 9.23 | 4.62 | 3.08 |
| Tonic Alertness | 27.63 | 19.74 | 7.89 | 11.84 |
| Phasic Alertness | 35.53 | 39.47 | 7.89 | 10.53 |
| Phonemic Fluency | 15.28 | 18.06 | .. | .. |
| Semantic Fluency | 13.89 | 11.11 | .. | .. |
| Processing Speed (TMT A) | 15.38 | 21.54 | .. | .. |
| Cognitive Flexibility (TMT B) | 24.62 | 21.54 | .. | .. |
| Cognitive Control (Stroop test) | 10.53 | 31.58 | 7.02 | 8.77 |

## **Table S3. Comparative analysis of statistically significant structural brain changes in matched healthy controls (HC) and post-COVID patients: Baseline and follow-up assessments with adjusted p-values and omega-squared effect sizes (ω²).**

| **Brain Regions** | **HC vs COVID (Baseline)**  *p-value* | **ω²** | **HC vs COVID (Follow-up)**  *p-value* | **ω²** | **Baseline vs Follow-up (COVID)**  *p-value* | **ω²** |
| --- | --- | --- | --- | --- | --- | --- |
| Right Amygdala | 0.14 | 0.93 | 0.21 | 0.89 | <.05* | 0.99 |
| Brain Stem | 0.15 | 0.92 | <.01** | 0.99 | <.001*** | 1.0 |
| Right Lateral Ventricle | 0.79 | 0.60 | <.05* | 0.98 | <.01** | 0.99 |
| Left Lateral Ventricle | <.001*** | 1.0 | <.05* | 0.98 | <.01** | 0.99 |
| Left Ventral Diencephalon | 0.22 | 0.88 | 0.40 | 0.79 | <.001*** | 1.0 |
| Right postcentral gyrus medial segment | <.001*** | 1.0 | <.01** | 1.0 | <.001*** | 1.0 |
| Left postcentral gyrus medial segment | 0.35 | 0.82 | <.01** | 1.0 | <.001*** | 1.0 |
| Right subcallosal area | 0.09 | 0.95 | 0.52 | 0.73 | <.05* | 0.99 |

## **Table S4. Comparative analysis of statistically significant functional brain regions: Baseline and follow-up in matched healthy Controls (HC) and post-COVID patients with adjusted p-values and omega-squared effect Sizes (ω²).**

| **Lobe** | **Brain Regions** | **HC vs COVID (Baseline)**  *p-value* | **ω²** | **HC vs COVID**  **(Follow-up)** *p-value* | **ω²** | **Baseline vs Follow-up (COVID)**  *p-value* | **ω²** |
| --- | --- | --- | --- | --- | --- | --- | --- |
| Limbic Lobe | Cingulate Gyrus, left caudodorsal area | 0.30 | 0.85 | 0.11 | 0.95 | **<.01**** | 0.99 |
|  | Cingulate Gyrus,  left subgenual area | 0.31 | 0.84 | 0.38 | 0.81 | **<.01**** | 0.98 |
| Temporal Lobe | Fusiform Gyrus,  left rostroventral area | 0.92 | 0.54 | 0.28 | 0.86 | **<.01**** | 0.98 |
|  | Fusiform Gyrus,  right lateroventral area | 0.73 | 0.63 | 0.78 | 0.61 | **<.01**** | 0.97 |
| Frontal Lobe | Inferior Frontal Gyrus,  right rostral area | 0.38 | 0.81 | 0.24 | 0.88 | **<.01**** | 0.99 |
|  | Inferior Frontal Gyrus,  right opercular area | 0.93 | 0.54 | **<.001***** | 1.00 | **<.01**** | 0.97 |
| Insular Lobe | Insular Gyrus,  left ventral agranular insula | 0.83 | 0.58 | 0.89 | 0.55 | **<.001***** | 0.99 |
|  | Insular Gyrus,  right ventral agranular insula | 0.72 | 0.64 | 0.37 | 0.81 | **<.05*** | 0.94 |
| Parietal Lobe | Inferior Parietal Lobule,  right rostrodorsal area | **<.001***** | 1.00 | **<.01**** | 1.00 | 0.86 | 0.75 |
|  | Inferior Parietal Lobule,  right caudal area | **<.05*** | 0.99 | 0.16 | 0.92 | 0.66 | 0.37 |
|  | Inferior Parietal Lobule,  left rostroventral area 39 | **<.001***** | 0.98 | 0.81 | 0.92 | **<.001***** | 0.98 |
|  | Inferior Parietal Lobule,  left rostroventral area 40 | 0.92 | 0.98 | 0.53 | 0.99 | 0.93 | 0.10 |
| Temporal Lobe | Inferior Temporal Gyrus,  left extreme lateroventral area | 0.41 | 0.75 | 0.26 | 0.93 | **<.05*** | 0.91 |
|  | Inferior Temporal Gyrus, left rostral area | 0.47 | 0.60 | 0.23 | 0.73 | **<.05*** | 0.98 |
|  | Inferior Temporal Gyrus, right rostral area | **<.05*** | 0.99 | 0.10 | 0.86 | **<.001***** | 0.98 |
|  | Inferior Temporal Gyrus,  Right caudolateral of area 20 | 0.68 | 0.91 | 0.37 | 0.99 | 0.053 | 0.96 |
| Occipital Lobe | Lateral Occipital Cortex,  left middle occipital gyrus | **<.05*** | 0.97 | 0.16 | 0.75 | **<.001***** | 0.99 |
|  | Lateral Occipital Cortex, right inferior occipital gyrus | **<.05*** | 0.99 | 0.32 | 0.81 | **<.05*** | 0.81 |
|  | Lateral Occipital Cortex,  left medial superior occipital gyrus | 0.57 | 0.97 | 0.23 | 0.94 | **<.05*** | 0.81 |
|  | Lateral Occipital Cortex, right medial superior occipital gyrus | **<.05*** | 1.00 | **<.05*** | 0.92 | **<.01**** | 0.97 |
|  | Lateral Occipital Cortex,  left lateral superior occipital gyrus | 0.49 | 0.81 | 0.13 | 0.79 | 0.64 | 0.10 |
|  | Lateral Occipital Cortex,  right lateral superior occipital gyrus | 0.06 | 0.71 | 0.07 | 0.98 | **<.05*** | 0.94 |
| Frontal Lobe | Middle Frontal Gyrus,  left lateral area | 0.24 | 0.70 | **<.05*** | 0.60 | 0.20 | 0.37 |
|  | Middle Frontal Gyrus,  right lateral area | **<.05*** | 0.89 | 0.55 | 0.84 | 0.07 | 0.98 |
| Temporal Lobe | Middle Temporal Gyrus,  right caudal area | 0.98 | 0.98 | 0.10 | 1.00 | **<.05*** | 0.196 |
|  | Middle Temporal Gyrus,  left rostral area | 0.81 | 1.00 | 0.54 | 0.98 | 0.61 | 0.37 |
|  | Middle Temporal Gyrus,  left dorsolateral area | **<.05*** | 0.97 | 0.29 | 1.00 | **<.05*** | 0.99 |
| Occipital Lobe | MedioVentral Occipital Cortex, left caudal cuneus gyrus | 0.18 | 0.80 | **<.05*** | 0.65 | **<.05*** | 0.98 |
|  | MedioVentral Occipital Cortex, right rostral lingual gyrus | 0.06 | 0.87 | 0.50 | 0.86 | 0.84 | 0.54 |
| Frontal Lobe | Orbital Gyrus,  left lateral area | 0.78 | 0.85 | 0.34 | 0.95 | 0.07 | 0.58 |
|  | Orbital Gyrus,  right lateral area | 0.89 | 0.84 | **<.001***** | 0.81 | 0.34 | 0.64 |
|  | Orbital Gyrus,  left medial area | **<.05*** | 0.54 | 0.39 | 0.86 | **<.001***** | 0.05 |
|  | Orbital Gyrus,  right medial area | **<.001***** | 0.63 | 0.13 | 0.61 | **<.001***** | 0.99 |
| Parietal Lobe | Precuneus,  left medial area | **<.01**** | 0.81 | 0.16 | 0.88 | **<.05*** | 0.98 |
| Temporal Lobe | Parahippocampal Gyrus, right rostral area | 0.62 | 0.54 | 0.21 | 1.00 | 0.11 | 0.39 |
|  | Parahippocampal Gyrus, left caudal area | 0.37 | 0.58 | 0.41 | 0.55 | **<.05*** | 0.97 |
|  | Parahippocampal Gyrus,  left entorhinal cortex | 0.58 | 0.64 | **<.05*** | 0.81 | **<.05*** | 0.99 |
|  | Parahippocampal Gyrus,  right temporal agranular insular cortex | 0.91 | 1.00 | 0.46 | 1.00 | **<.05*** | 0.97 |
| Parietal Lobe | Postcentral Gyrus,  left upper limb, head and face region | 0.61 | 0.99 | 0.81 | 0.92 | **<.05*** | 0.99 |
|  | Postcentral Gyrus, right tongue and larynx region | 0.22 | 0.98 | 0.31 | 0.92 | 0.11 | 0.46 |
|  | Postcentral Gyrus, right trunk region | 0.58 | 0.98 | **<.05*** | 0.99 | **<.05*** | 0.94 |
| Frontal Lobe | Precentral Gyrus, left caudal dorsolateral area | 0.41 | 0.75 | 0.30 | 0.93 | 0.11 | 0.75 |
|  | Precentral Gyrus,  left trunk region | **<.001***** | 0.60 | 0.83 | 0.73 | 0.24 | 0.37 |
|  | Precentral Gyrus,  right trunk region | **<.05*** | 0.99 | 0.19 | 0.86 | **<.05*** | 0.98 |
|  | Precentral Gyrus, right caudal ventrolateral area | 0.40 | 0.91 | 0.80 | 0.99 | **<.001***** | 0.94 |
|  | Superior Frontal Gyrus, right lateral area | **<.05*** | 0.97 | **<.001***** | 0.75 | 0.053 | 0.75 |
|  | Superior Frontal Gyrus, left medial area | 0.52 | 0.99 | 0.57 | 0.81 | **<.05*** | 0.89 |
| Parietal Lobe | Superior Parietal Gyrus, right rostral area | **<.01**** | 1.00 | **<.05*** | 0.94 | 0.57 | 0.75 |
|  | Superior Parietal Gyrus, left intraparietal area | 0.07 | 0.71 | **<.001***** | 0.92 | **<.05*** | 0.84 |
| Subcortical Nuclei | Thalamus,  right pre-motor thalamus | 0.40 | 0.81 | 0.71 | 0.79 | **<.01**** | 0.98 |
|  | Thalamus, left rostral temporal thalamus | 0.27 | 0.71 | 0.29 | 0.98 | **<.001***** | 0.99 |

# **3. Figures**

**Figure S1. Overview of symptom evolution.** Shown is the proportion and evolution of individual symptoms at the follow-up visit.

**
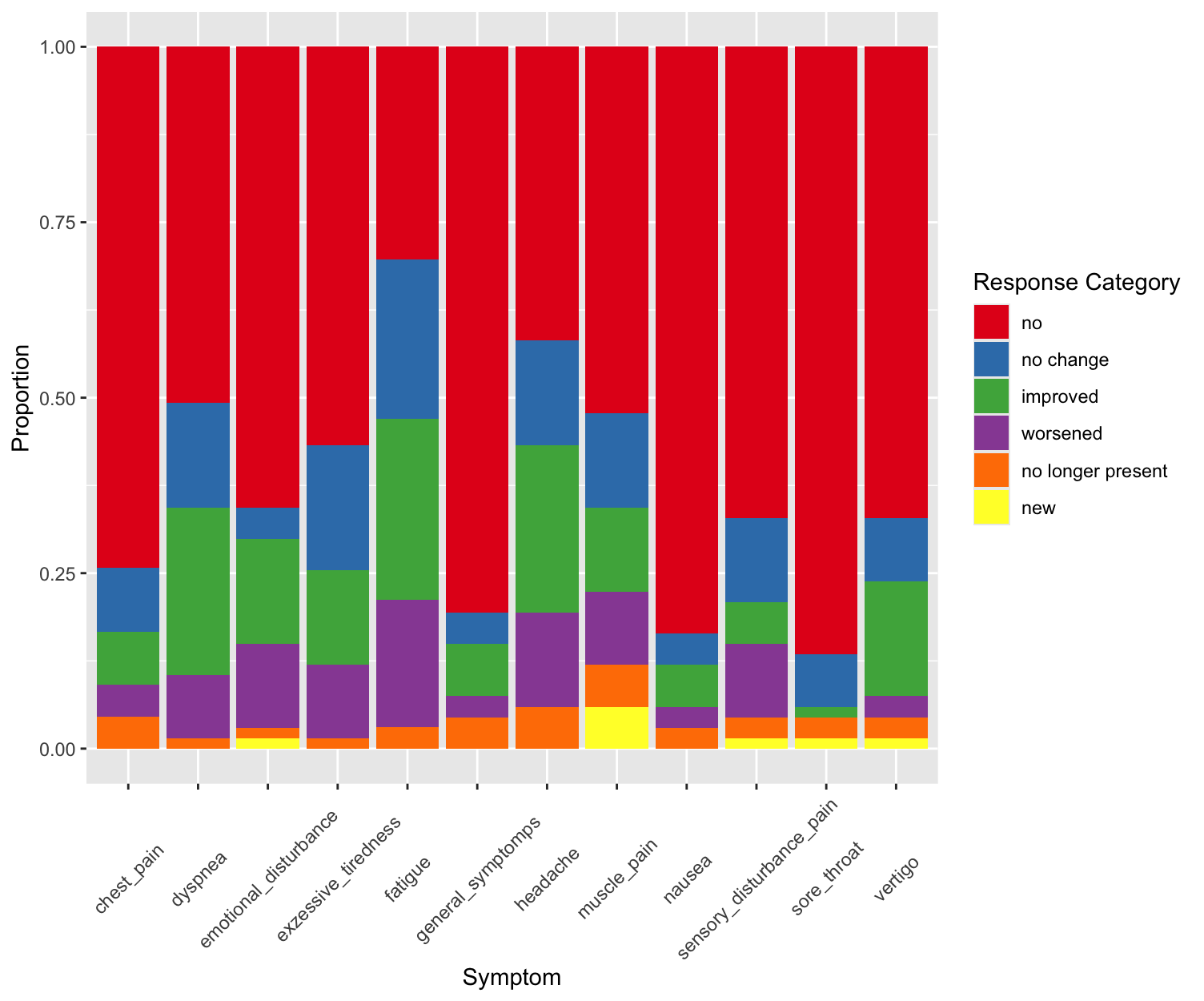
**

# **4. References**

Andersson, J. L. R., Skare, S., & Ashburner, J. (2003, 2003/10/01/). How to correct susceptibility distortions in spin-echo echo-planar images: application to diffusion tensor imaging. *Neuroimage, 20*(2), 870-888. <https://doi.org/https://doi.org/10.1016/S1053-8119(03)00336-7>

Avants, B. B., Epstein, C. L., Grossman, M., & Gee, J. C. (2008, 2008/02/01/). Symmetric diffeomorphic image registration with cross-correlation: Evaluating automated labeling of elderly and neurodegenerative brain. *Medical Image Analysis, 12*(1), 26-41. <https://doi.org/https://doi.org/10.1016/j.media.2007.06.004>

Behzadi, Y., Restom, K., Liau, J., & Liu, T. T. (2007, Aug 1). A component based noise correction method (CompCor) for BOLD and perfusion based fMRI. *Neuroimage, 37*(1), 90-101. <https://doi.org/10.1016/j.neuroimage.2007.04.042>

Ciric, R., Wolf, D. H., Power, J. D., Roalf, D. R., Baum, G. L., Ruparel, K., Shinohara, R. T., Elliott, M. A., Eickhoff, S. B., Davatzikos, C., Gur, R. C., Gur, R. E., Bassett, D. S., & Satterthwaite, T. D. (2017, Jul 1). Benchmarking of participant-level confound regression strategies for the control of motion artifact in studies of functional connectivity. *Neuroimage, 154*, 174-187. <https://doi.org/10.1016/j.neuroimage.2017.03.020>

Cox, R. W., & Hyde, J. S. (1997, Jun-Aug). Software tools for analysis and visualization of fMRI data. *NMR Biomed, 10*(4-5), 171-178. <https://doi.org/10.1002/(sici)1099-1492(199706/08)10:4/5><171::aid-nbm453>3.0.co;2-l

Dale, A. M., Fischl, B., & Sereno, M. I. (1999, 1999/02/01/). Cortical Surface-Based Analysis: I. Segmentation and Surface Reconstruction. *Neuroimage, 9*(2), 179-194. <https://doi.org/https://doi.org/10.1006/nimg.1998.0395>

Fonov, V. S., Evans, A. C., McKinstry, R. C., Almli, C. R., & Collins, D. L. (2009, 2009/07/01/). Unbiased nonlinear average age-appropriate brain templates from birth to adulthood. *Neuroimage, 47*, S102. <https://doi.org/https://doi.org/10.1016/S1053-8119(09)70884-5>

Greve, D. N., & Fischl, B. (2009, Oct 15). Accurate and robust brain image alignment using boundary-based registration. *Neuroimage, 48*(1), 63-72. <https://doi.org/10.1016/j.neuroimage.2009.06.060>

Jenkinson, M., Bannister, P., Brady, M., & Smith, S. (2002, Oct). Improved optimization for the robust and accurate linear registration and motion correction of brain images. *Neuroimage, 17*(2), 825-841. <https://doi.org/10.1016/s1053-8119(02)91132-8>

Klein, A., Ghosh, S. S., Bao, F. S., Giard, J., Hame, Y., Stavsky, E., Lee, N., Rossa, B., Reuter, M., Chaibub Neto, E., & Keshavan, A. (2017, Feb). Mindboggling morphometry of human brains. *PLoS Comput Biol, 13*(2), e1005350. <https://doi.org/10.1371/journal.pcbi.1005350>

Lanczos, C. (1964). Evaluation of Noisy Data. *Journal of the Society for Industrial and Applied Mathematics, Series B Numerical Analysis 1 (1): 76–85*. <https://doi.org/https://doi.org/10.1137/0701007>.

Patriat, R., Reynolds, R. C., & Birn, R. M. (2017, Jan 1). An improved model of motion-related signal changes in fMRI. *Neuroimage, 144*(Pt A), 74-82. <https://doi.org/10.1016/j.neuroimage.2016.08.051>

Power, J. D., Mitra, A., Laumann, T. O., Snyder, A. Z., Schlaggar, B. L., & Petersen, S. E. (2014, Jan 1). Methods to detect, characterize, and remove motion artifact in resting state fMRI. *Neuroimage, 84*, 320-341. <https://doi.org/10.1016/j.neuroimage.2013.08.048>

Satterthwaite, T. D., Elliott, M. A., Gerraty, R. T., Ruparel, K., Loughead, J., Calkins, M. E., Eickhoff, S. B., Hakonarson, H., Gur, R. C., Gur, R. E., & Wolf, D. H. (2013, Jan 1). An improved framework for confound regression and filtering for control of motion artifact in the preprocessing of resting-state functional connectivity data. *Neuroimage, 64*, 240-256. <https://doi.org/10.1016/j.neuroimage.2012.08.052>

Tustison, N. J., Avants, B. B., Cook, P. A., Zheng, Y., Egan, A., Yushkevich, P. A., & Gee, J. C. (2010). N4ITK: Improved N3 Bias Correction. *IEEE Transactions on Medical Imaging, 29*(6), 1310-1320. <https://doi.org/10.1109/TMI.2010.2046908>

Zhang, Y., Brady, M., & Smith, S. (2001). Segmentation of brain MR images through a hidden Markov random field model and the expectation-maximization algorithm. *IEEE Transactions on Medical Imaging, 20*(1), 45-57. <https://doi.org/10.1109/42.906424>
